# Permutation Entropy and Signal Energy Increase the Accuracy of Neuropathic Change Detection in Needle EMG

**DOI:** 10.1155/2018/5276161

**Published:** 2018-01-24

**Authors:** O. Dostál, O. Vysata, L. Pazdera, A. Procházka, J. Kopal, J. Kuchyňka, M. Vališ

**Affiliations:** ^1^Faculty of Medicine and University Hospital Hradec Králové, Charles University in Prague, Sokolská Street 581, 500 05 Hradec Králové, Czech Republic; ^2^Department of Computing and Control Engineering, Institute of Chemical Technology, Technická 5, 166 28 Prague 6, Czech Republic; ^3^Neurocenter Caregroup, Ltd., Jiráskova 1389, Rychnov nad Kněžnou, Czech Republic

## Abstract

*Background and Objective. *Needle electromyography can be used to detect the number of changes and morphological changes in motor unit potentials of patients with axonal neuropathy. General mathematical methods of pattern recognition and signal analysis were applied to recognize neuropathic changes. This study validates the possibility of extending and refining turns-amplitude analysis using permutation entropy and signal energy.* Methods.* In this study, we examined needle electromyography in 40 neuropathic individuals and 40 controls. The number of turns, amplitude between turns, signal energy, and “permutation entropy” were used as features for support vector machine classification.* Results.* The obtained results proved the superior classification performance of the combinations of all of the above-mentioned features compared to the combinations of fewer features. The lowest accuracy from the tested combinations of features had peak-ratio analysis.* Conclusion.* Using the combination of permutation entropy with signal energy, number of turns and mean amplitude in SVM classification can be used to refine the diagnosis of polyneuropathies examined by needle electromyography.

## 1. Introduction

Qualitative visual analysis of MUPs and interference patterns may be useful for diagnosis when there are clear changes, but this approach may be misleading in patients with more subtle lesions [1]. Computational processing helps clinicians draw conclusions from large data sets, such as complex waveforms acquired from EMG. Performing single motor unit potential (MUP) analysis during a weak muscle contraction a is time-consuming test. For some of the examined subjects, it is difficult to maintain a constant weak contraction. The ideal solution for a description of the EMG signal would be perfect decomposition of the action potentials of motor units to determine an interference curve. In clinical practice, the precise decomposition of intramuscular EMG signals still has limited applications. In most cases, the signal is not fully decomposed or only a few representative action potentials are collected. One way of quantifying the electromyographic interference pattern is by measuring the number of turns and the mean amplitude change between successive turns. A turn occurs at a peak at which the signal changes direction and differs by at least 100 *μ*V in amplitude from the previous and following turns [2]. A disadvantage of the Willison analysis is that it does not appear to be as sensitive as single MUP analysis. In axonal polyneuropathy, there is a loss of motor units, which leads to simplification of EMG curves. Therefore, reductions in signal entropy and energy are expected. While muscle contraction increases, more motor units are firing. This leads to an increase in signal entropy.

The aim of this study is to compare the performance of classification based on “turns-amplitude” analysis with classification derived from an extended number of features, including “permutation entropy” and signal energy. Subsequently, a supervised learning method called the “support vector machine” is used for binary classification of the data.

## 2. EMG Data Acquisition

### 2.1. Data Acquisition

This study focuses on an analysis of needle EMG signals from 40 reference and 40 neuropathic individuals. All signals were acquired with the sampling frequency* Fs* = 12,5 kHz during voluntary muscle contraction lasting 4 seconds. The maximum force in the tibialis anterior muscle was measured before EMG needle insertion by a dynamometer. Electrical activity during 30% muscle contraction was recorded by a concentric needle electrode with a leading-off area of 0.07 mm^2^. The filter setting was in a range between 5 Hz and 10 kHz with an amplitude setting from the 100 *μ*V/division to 2 mv/division and a sweep speed of 10 ms/division.

Motor nerve conduction studies (NCS) of median and peroneal nerves, sensory NCS of median and sural nerves, and needle EMG from the tibialis anterior muscle were performed using a standard technique with an Alien EMG device. Additional nerve conduction testing was performed as indicated by the pattern and severity of abnormal findings to determine sensory, motor, axonal, and demyelinating features of the polyneuropathy. The study was approved and supervised by the Local Ethics Committee.

### 2.2. Data Set

The normal controls (18–64 years old) included 40 patients examined for paraesthesias of central origin (sclerosis multiplex), restless legs syndrome, and gait instability with no neuropathic problems. The neuropathy set involved 40 patients (19–74 years old) diagnosed with polyneuropathy based on a combination of clinical signs, neuropathic symptoms, and electrodiagnostic findings as established by the American Association of Neuromuscular Electrodiagnostic Medicine [3]. This set included patients with a history of chronic alcohol abuse, with a history of chemotherapy (vincristine, paclitaxel), and with hereditary motor sensory neuropathy.

## 3. Data Processing

### 3.1. Signal Analysis

Values obtained by evaluating the properties of EMG waveforms were used as inputs for machine learning to sort the data into two groups. Mathematical methods were studied and proposed for the estimation of characteristic features of an EMG signal *x*(*n*)_*n*=1_^*N*^ of length *N* obtained with a sampling frequency *Fs* or sampling time *Ts* = 1/*Fs* which included the following calculations.

#### 3.1.1. Turns and Amplitude Analysis

TA analysis is a widely used method of interference pattern analysis developed by Willison in the 1960s. The principle is to compare the number of turns over time that are defined as positive or negative potential changes greater than a selected threshold (usually 100 *μ*V).

The Willison rate is defined by the relation *W*_rate_ = (1/*T*_*s*_*N*)(∑_*k*=1_^*N*−1^*f*(*k*)), where (*f*(*k*) = *d*(*k*) > THRESH)_*k*=1_^*N*−1^ forms a sequence for differences (*d*(*k*) = |*x*(*k* + 1) − *x*(*k*)|)_*k*=1_^*N*−1^ [4–6].

Fuglsang–Frederiksen used the ratio of the number of turns per second to the mean amplitude (peak-ratio method) to distinguish myopathies from neuropathic disorders [4, 7]. In neuropathic subjects, the sensitivity of this method approaches the sensitivity in MUAP analysis, whereas in myopathic subjects it even exceeds it [8]. The threshold in our study was set to 100 *μ*V. An amplitude was measured between successive turns. A turn was defined as a change in the direction of the signal of at least 100 microvolts.

#### 3.1.2. Permutation Entropy

Permutation entropy (PE) is a way of quantifying the complexity of data, which was introduced in 2002 by Bandt and Pompe [9]. Unlike former entropy approaches, PE has significant advantages, particularly in time series (i.e., robustness, lower computational requirements, and easy calculation for chaotic and noisy time series). The idea is to select all possible data sequences of length *n* (the order of permutation) and compare them with all possible permutation patterns *π*_1_*–π*_*n*!_ of* n *members that represent the rank orders of data values. Apart from pattern length *n*, there is a second parameter time lag (*τ*) that describes the time delay between successive patterns (to avoid error in data with a high frequency of equal values). Based on the occurrence of permutation patterns within the data set, a PE is calculated according to

(1) $$\begin{array}{c} H_{n}=-{\sum_{j=1}^{}\;}^{n!}\left(\;p\left(\;\pi \;\right)\left(\;\log2\left(\;p\left(\;\pi \;\right)\;\right)\;\right)\;\right), \end{array}$$

where *p*(*π*) stands for the relative frequencies of possible permutation patterns. To be able to compare entropies with different *n*, the following relation is defined [9, 10]:

(2) $$\begin{array}{c} h_{n}=\frac{H\left(n\right)}{\left(n-1\right)}. \end{array}$$

For the purposes of our study, the order of permutation (*n*) and the time lag (*τ*) were set to 3 or 2, respectively, to allow for the same rank for equal values.

#### 3.1.3. Signal Energy

The signal energy is defined as the sum of the absolute values of the samples per second.

### 3.2. Data Classification: Support Vector Machine

**Figure 1:**
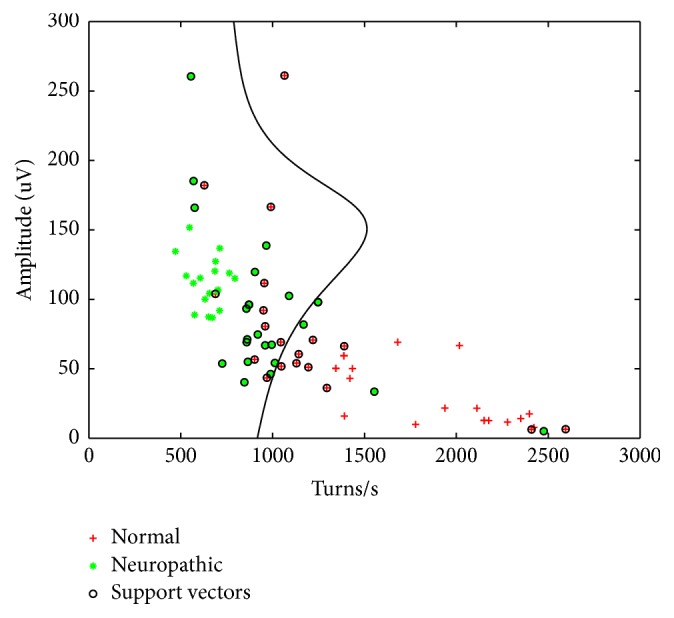
“Turns-amplitude” analysis.

This method was developed in the 1960s when Vapnik introduced an algorithm for linear binary separation of a data set that works on a training set in which each data point *x*_*i*_ is given information about its classification (−1 or 1) [11]. Data are separated by a hyperplane that is constructed so that it has a maximum distance from the nearest points of both groups of data (the support vectors), which creates the widest possible zone (margin) where no data points occur. In case the data are not linearly separable (e.g., if no such hyperplane exists), we can use the “soft margin method” in which some data points are accepted as errors. A slack variable is introduced to determine the trade-off between margin maximization and training error minimization [12]. In 1992, Boser, Guyon, and Vapnik created a method for nonlinear classification by applying a kernel trick that transforms the data to a higher dimension where they can be linearly separated [13]. Projection of the hyperplane from high dimensional space into two dimensions is depicted as a nonlinear curve that efficiently separates the data points. Commonly used kernels include homogenous and inhomogeneous polynomial, Gaussian radial basis function, and hyperbolic tangent [14, 15]. The support vector machine is a powerful tool for binary classification of data, which was successfully applied in various branches ranging from handwritten digits and face recognition to bioinformatics such as interpretation of DNA expression [13, 14]. Another modification of this approach can be used for regression analysis or for clustering of the data into groups (unsupervised learning with unlabeled data points) [16, 17]. Here, a cross-validated SVM classifier was optimized using Bayesian optimization. The radial basis kernel function was selected for separation of the data. Parameters for balancing the error and margin width were optimized by quadratic programming. For binary classification, two feature vectors were used. The Gaussian radial basis function kernel had a scaling factor 1. The turns-amplitude classifier was compared to turns-amplitude-entropy classifier, turns-amplitude-energy classifier, and turns-amplitude-entropy-energy classifier, as shown in Figure 1 and Table 2.

### 3.3. Performance Evaluation

To assess the performance of SVM with different parameters, we used the following measures: the number of true positives (TP), the number of false positives (FP), the number of true negatives (TN), the number of false negatives (FN), sensitivity of positive examples (Sn^+^), specificity of positive examples (Sp^+^), sensitivity of negative examples (Sn^−^), specificity of negative examples (Sp^−^), accuracy (Ac), and the Matthews correlation coefficient (MCC). These measures can be defined as follows [18]:

(3) Sn+=TPTP+FN,Sp+=TPTP+FP,Sn−=TNTN+FP,Sp−=TNTN+FN,MOC=TP×TN−FP×FNTP+FN×TP+FP×TN+FN×TN+FP,TPR=TPTP+FN,FPR=FPFP+TN.

## 4. Results

Between the two groups, the differences in mean values of all parameters were statistically significant (Table 1). The accuracy of the turns-amplitude analysis was the lowest, whereas a combination of all parameters had the highest accuracy (Table 2).

## 5. Discussion

Permutation entropy is used to quantify the level of order in EMG signals, while the peak-ratio method and energy express the statistical properties of the signal. These parameters are therefore mutually independent and can be combined to achieve higher accuracy. In addition to the process of waveform simplification after the loss of motor units, there is also a formation process of large and complex motor units during reinnervation. Surprisingly, this second process has caused an increase in entropy in the resulting EMG curve. Loss of motor units may also be compensated for by an increase in the firing frequency. However, this mechanism should not lead to an increase in entropy because it is a repetition of the same pattern. Another reason for the limited entropy benefit is the fact that the group of polyneuropathic patients includes subjects with various degrees of axonal loss. Significant alterations in the number of changes and morphological changes of motor units do not have to be present for moderate impairments to arise.

## 6. Conclusion

Although the combination of permutation entropy and signal energy with the peak-ratio method significantly improves accuracy in classifying axonal polyneuropathy, the payoff of using this methodology is limited. In terms of entropy, there are probably two contradictory processes that lead to the loss of motor units and the emergence of complex reinnervation potentials.

## Figures and Tables

**Table 1:** Characteristics of the parameters used for SVM training.

Parameter	Normal (mean ± SD)	Neuropathic (mean ± SD)	*p* value
Number of turns/s	1496.50 ± 557.33	838.40 ± 345.67	1.33*∗*10^−8^
Interspike amplitude (*μ*V)	59.30 ± 52.21	99.99 ± 44.91	3.54*∗*10^−4^
Energy (mV/sec)	479.57 ± 199.20	304.37 ± 276.75	1.70*∗*10^−3^
Entropy	6.85 ± 0.70	7.25 ± 0.97	3.85*∗*10^−2^

**Table 2:** Comparison of the SVM classifier with different parameters for performance measures (all the SVM classifiers were trained with a set of default parameters; see SVM under Data Processing). A leave-one-out cross-validation was performed for each result.

Performance measure	Turns, amplitude	Turns, amplitude, entropy	Turns, amplitude, energy	Turns, amplitude, entropy, energy
TP	35.13	33.18	33.63	36.10
FN	3.88	5.83	5.38	2.90
FP	9.88	7.68	7.23	4.95
TN	29.13	31.33	31.78	34.05
Sn+ (%)	0.90	0.85	0.86	0.93
Sp+ (%)	0.78	0.81	0.82	0.88
Sn− (%)	0.75	0.80	0.81	0.87
Sp− (%)	0.88	0.84	0.86	0.92
Ac (%)	0.82	0.83	0.84	0.90
MCC	0.66	0.65	0.68	0.80
